# Does the new cooperative medical scheme reduce inequality in catastrophic health expenditure in rural China?

**DOI:** 10.1186/s12913-016-1883-7

**Published:** 2016-11-14

**Authors:** Na Guo, Tor Iversen, Mingshan Lu, Jian Wang, Luwen Shi

**Affiliations:** 1 School of Pharmaceutical Sciences, Peking University, Beijing, China; 2 International Research Center for Medicinal Administration, Peking University, Beijing, China; 3 Department of Health Management and Health Economics, University of Oslo, Oslo, Norway; 4 Department of Economics, University of Calgary, 2500 University Drive NW, Calgary, AB T2N 1N4 Canada; 5 School of Public Health, Shandong University, Jinan, China

**Keywords:** New cooperative medical scheme, Inequality, Catastrophic health expenditures, Concentration index

## Abstract

**Background:**

In 2003, the New Cooperative Medical Scheme (NCMS) was introduced in China to re-establish health insurance for the country’s vast rural population. In addition, the coverage of NCMS has been expanding after the new health care reform launched in 2009. This study aims to examine whether the NCMS and its recent expansion have reached the goal of reducing the risk and inequality of catastrophic health spending for rural residents in China.

**Methods:**

We conducted a face-to-face household survey in three counties of the Shandong province in 2009 and 2012. Using this unique panel data, we examined the changes in the incidence and intensity of catastrophic health expenditures (CHEs) before and after NCMS reimbursement. We used concentration index (CI) and decomposition method to study the changes in inequality in CHEs.

**Results:**

We found that NCMS reimbursement played a role of reducing both the incidence and intensity of CHEs, and that this impact was stronger after the new health care reform was launched. After reimbursement, the concentration indices for CHEs were 0.073 and 0.021 in 2009 and 2012, indicating that the rich had a greater tendency to incur CHEs and there existed less inequality in the incidence of CHEs after reimbursement in 2012 compared with 2009. The decomposition analysis results suggested that changes in CHE inequality between 2009 and 2012 were attributed to changes in economic status and household size rather than reimbursement levels.

**Conclusions:**

Our results indicated that inequality was shrinking from 2009 to 2012, which could be a result of fewer rich people having CHEs in 2012 compared with 2009. The impact of NCMS in alleviating the financial burden of rural residents was still limited, especially among the poor. Health care reform policies in China that aim to reduce CHEs must continue to place an emphasis on improving reimbursement, cost containment, and reducing income inequalities.

## Background

Catastrophic health expenditures (CHEs) can induce households into poverty and is a major concern in many countries [1–3]. CHEs refer to high levels of healthcare expenditures that affect a household’s standard of living by causing the household to sacrifice other basic expenditures to pay for health costs [4]. CHEs can be defined as health payments that exceed a fixed threshold share of household income or total expenditures in a given period, usually one year [5]. Some studies define CHEs as total health expenditures that exceed 10 % of total annual household income [3, 6], and others operate with a limit of 40 % when capacity to pay (CTP) is used as the denominator [7–11].

Health insurance is implemented as a measure to protect households against CHEs and to improve access to medical care. For example, results from a 2008 study showed that a universal coverage policy that was introduced in Thailand contributed to preventing financial catastrophe and impoverishment due to reduced out-of-pocket health care payments [12]. A 2007 study from India indicated that community health insurance appeared to be effective in protecting members against CHEs among hospitalised patients [6]. Wagstaff et al. analysed three surveys and suggested that previous health insurance reforms in China led to increases in out-of-pocket spending and the risk of large, catastrophic expenses [1]. The existing literature suggests that the effect of health insurance on preventing CHE is partial or limited. In countries where out-of pocket expenditures are the most important source of health care financing, their effects on household economic status can be severe, particularly among the poor.

Prior to 1978, the majority of the rural population in China was covered by the Cooperative Medical Scheme (CMS), a collective-economy, prepaid health security program. However, under the Chinese economic reform that began in 1978, the CMS collapsed, leaving the vast majority of the rural population with no insurance coverage. Since the Chinese economic reform, medical expenditures have been a major financial burden for many rural households in China [13–15]; and access to and utilisation of health care in rural areas was governed by the ability to pay. Recognising the magnitude of this issue, the Chinese government initiated the New Cooperative Medical System (NCMS) in 2003 to re-establish health insurance system for the rural population. It was advocated for, organised, and sponsored by the government, with rural residents’ voluntary enrolment. One of its explicit goals is to effectively reduce rural people’s financial burden for health services and to relieve impoverishment by preventing catastrophic health expenditures (CHEs). The research findings on whether the NCMS achieved the objective in reducing CHEs are mixed. Some studies suggested that NCMS reimbursement helped relieve CHEs to a certain degree, promoted equity in health financing [3, 16], and played a certain role in reducing out of pocket health expenditures [17]. Other studies found that although the NCMS coverage rate is high, the impact is limited. For instance, Lei et al. found that out-of-pocket expenditures did not decrease [18]. Wagstaff et al. found that the NCMS increased utilisation of outpatient and inpatient services, but had not reduced the incidence of catastrophic out-of-pocket payments [19]. In 2010, Zhang et al. analysed data for 2004 and 2007 and found that the current NCMS program did not appear likely to reduce the incidence of CHEs [20]. Because of the variations in research periods and districts, there still is not sufficient and clear evidence to confirm the effects of the NCMS on alleviating catastrophic health expenditures in China [21].

To provide the population with convenient and affordable health care services, reduce the burden of medical costs, and relieve the problem of “difficult and costly access to health care services”, the Chinese government implemented the new Health Care Reform in 2009, with the first step in effect from 2009 to 2011. Under this reform, expanding NCMS coverage and improving the level of reimbursement was one of the most important policies. As a result, the coverage rate of NCMS reached over 97 %, and the annual benefit went to 1.315 billion people in 2011. From 2008 to 2011, financial subsidies at all levels for NCMS have improved from 120 yuan (about 18.80 USD) to 200 yuan (about 31.34 USD) per person per year [22]. However, there is still insufficient evidence of whether the increase in government contribution and expansion of NCMS coverage has reduced financial burden [23, 24].

Therefore, in 2007, we conducted a face-to-face household survey of rural residents in three counties in the Shandong province, where the NCMS was piloted. Some of the households in the original survey were interviewed again in follow-up surveys first in 2009 and then in 2012. Using this unique panel data collected from the same households before and after the new Health Care Reform, our study aims to fill the gap by contributing to the evidence of the effects of the NCMS after the implementation of the new Health Care Reform. To the best of our knowledge, this is the first study to utilise panel data to analyse the impacts of the NCMS on CHEs before and after the new Health Care Reform.

The paper is structured as follows: Section 2 contains data and descriptive statistics; Section 3 explains the methods used in the analyses; Section 4 presents results; Section 5 offers a discussion of the study’s results; and Section 6 presents a brief conclusion.

## Data and description

### Sampling method and sample selection

We conducted a field investigation on the NCMS in the Shandong province that entailed a face-to-face survey, originally in 2007 and then in 2009 and 2012.

Three stratifications from the counties that implemented the NCMS in Jinan, Shandong, were chosen based on per capita net family income of the rural area into high, middle, and low using a stratified random sampling method. Then, one county was chosen from each stratification. As a result, three counties in Jinan City were included in the survey: Zhangqiu, Changqing, and Pingyin. These three counties are representative of the initial pilot counties in Shandong province in 2005 in the following ways: Zhangqiu is a high-income county with per capita net family income of rural area 11,965.3 yuan (about 1,875.44 USD); Changqing is a middle-income county with per capita net family income of rural area 10,079.1 yuan (about 1,579.80 USD); and Pingyin is a low-income county with per capita net family income of rural area 8,490.0 yuan (about 1,330.72 USD).

In the 2007 household survey, six towns were randomly selected in accordance with high, middle, and low incomes as measured by per capita net family income of the rural area in each selected county. Then, six villages were randomly selected as high, middle, and low incomes in each selected town. Finally, 30 households in each village were chosen using an equal distance sampling method and the village household registration lists; all household members were interviewed. However, the household head answered the questions in the absence of any members who were not home. This generated a sample of 3,240 households and 11,543 individuals.

In the 2009 household survey, due to a sudden budget cut, only three towns were surveyed among the six in each county that were included in the 2007 survey. The choice of the three towns was based on high, middle, and low incomes. The three towns selected were among the villages from 2007 survey were kept. Finally, 20 households were randomly selected from 30 households in each village that were included in the 2007 survey. The final sample comprised 985 households and 3,698 individuals.

In 2012, the same towns, villages, and households in the original 2007 survey were surveyed again. If a relevant household member was not home, the home was eliminated rather than our choosing another household. This resulted in a total of 2,174 households that comprised 8,110 individuals in our 2012 survey.

In this study, we only use the 2009 and 2012 surveys to compare the impacts of the NCMS on catastrophic health expenditures before and after reform. There are two reasons for not using the original 2007 survey data: first, the health care reform was implemented in 2009, and second, the quality of the 2007 data was not considered sufficient because the hospital expenses were cited by personal recall rather than based on receipts and the data was not reconfirmed by using the NCMS information system at each county. Because the NCMS coverage in these two years was very high, we kept only the NCMS enrolees. The valid sample size for this study was 3,534 and 7,707 individuals in the 2009 and 2012 waves, respectively. For this study, we used only the unbalanced panel data based on the year 2009. That is, the sample included all of the individuals in 2009 (3,534 individuals in 971 households) and the same individuals who were surveyed again in 2012 (2358 individuals in 694 households). Individuals in developing countries generally do not visit a health care provider unless they perceive themselves as either ill or injured. If there are unobserved factors correlated with the perception of illness and the likelihood of visiting a provider, then the coefficients in the equation will be biased. The biases can, therefore, be referred to as unobserved heterogeneity biases. As a result, we used panel data to analyse the hospital utilisation of households to account for unobserved heterogeneity at the household level and to be better able to capture the specific nature of the dependent variables. Descriptive statistics for the sample are shown in Table 2. However, when we measured the effects of the NCMS on the catastrophic health expenditures induced by hospitalisation, we only kept the households that had experienced hospitalisation in the year (2009 and 2012). After omitting the households with incomplete information, specifically, the lack of important data, such as expenditures, 131 and 108 households in 2009 and 2012, respectively, remained for further analysis of CHEs.

The survey was conducted by students at Shandong University. Before the survey all interviewers were trained by the principal investigator who designed this survey, and all interviewers practiced interviewing. During the survey, the interviewers conducted the household survey and face-to-face interviews with the individuals who agreed to participate. We also had two coordinators for each village who were familiar with the status of the fields to improve the response rate, and one supervisor who controlled the survey quality. The completeness of the questionnaires was checked by the supervisor of each village at the end of every day. If there was missing information on the survey, individuals would be telephoned and re-surveyed if possible. Ethical approval is not required for conducting this type of health services survey in China. However, our interviewers were instructed to obtain consent from all individuals when conducting the household survey and face-to-face interviews. The individuals were informed about their right to refuse to answer any question.

### The NCMS in sample districts

The development of the NCMS in the Shandong province follows the same trend as that across China. In general, in the Shandong province, all levels of government subsidised no less than 60 yuan (about 9.40 USD) and 200 yuan (about 31.34 USD) for the NCMS in 2009 and 2012, respectively. In the sample districts, in 2009, the total contribution to the NCMS was 70 yuan (about 10.97 USD) in Zhangqiu and Changqing counties. The central and local governments at all levels subsidised 60 yuan (about 9.40 USD) per farmer, and the farmer paid the remaining 10 yuan (about 1.57 USD) annually to enrol in the NCMS. In Pingyin County, the total contribution to the NCMS was 40 yuan (about 6.27 USD). The total subsidy from all levels of government was 30 yuan (about 4.70 USD) per farmer, with the farmer paying the remaining 10 yuan (about 1.57 USD) annually to enrol in the NCMS. In 2012, the total contributions were 250 yuan (about 39.18 USD) in each of these three counties. The government at all levels subsidised 200 yuan (about 31.34 USD) per farmer, with the farmer paying 50 yuan (about 7.84 USD) annually. The level of reimbursement largely increased in 2012 compared with 2009. The reimbursement rate for hospital costs also largely increased at all hospital levels in 2012. The lower-level hospitals had higher reimbursement rates than the higher-level hospitals in both years. The ceiling on the amount of annual reimbursement also increased in these three counties in 2012 compared with 2009 (Table 1).

**Table 1:** Descriptive statistics for NCMS policies and coverage in 2009 and 2012

	2009			2012		
	Zhangqiu	Changqing	Pingyin	Zhangqiu	Changqing	Pingyin
Deductible (yuan)						
Township hospital	0 (0 USD)	0 (0 USD)	0 (0 USD)	300 (47.01 USD)	200 (31.34 USD)	200 (31.34 USD)
County-level hospital	1,000 (156.7 USD)	0 (0 USD)	0 (0 USD)	400 (62.68 USD)	400 (62.68 USD)	400 (62.68 USD)
Ceiling (yuan)	35,000 (5,484.5 USD)	50,000 (7,835 USD)	20,000 (3,134 USD)	100,000 (15,670 USD)	120,000 (18,804 USD)	100,000 (15,670 USD)
Reimbursement percent (%)						
Township hospital	30	55	40	70	80	70
County-level hospital	25	22.5	40	55	45	55
Above county hospital	15	10	40	40	35	40
NCMS participation *N* (%)						
NCMS enrolees	3,558 (96.21)			7,881 (97.18)		
Non-enrolees	140 (3.79)			229 (2.82)		

Note: The reimbursement rate was calculated based on expenditures under 10,000 yuan (about 156.7 USD) based on NCMS policies

### Descriptive statistics

Information collected in the survey included sex, age, education level, occupation, health insurance, and health status, household income and expenditures, household size, health service utilisation (inpatient care with the recall period of one year for payments preceding the survey) and medical expenses. Sample descriptive statistics for the key variables for both 2009 and 2012 are provided in Table 2. The economic status was divided into five equal intervals based on household per capita expenditures. Age was divided into two groups (plus the reference group), household members 65 or over and members age 5 or under because the inpatient rates for these two groups are relatively high according to other studies [25–27]. The sex of the household head as identified by the household register used female as the reference; the education levels were less than primary school, junior middle school, and senior middle school, and above; occupation was farmer or non-farmer; household size was separated into two groups, 1 to 3 members and 4 members or more; the three self-assessed health status options were worse than normal (reference), normal, and better than normal.

**Table 2:** Descriptive statistics for study variables in 2009 and 2012

Variables	2009	2012
	*N* (%)	*N* (%)
Economic status (reference group = Quintile1)		
Quintile2	194 (19.98)	139 (20.03)
Quintile3	194 (19.98)	138 (19.88)
Quintile4	194 (19.98)	139 (20.03)
Quintile5	194 (19.98)	139 (20.03)
Household member ≥ 65	256 (26.36)	223 (32.13)
Household member ≤ 5	194 (19.98)	96 (13.83)
Household head		
Sex (male)	890 (91.66)	641 (92.36)
Education (reference group = below primary school level)		
Junior middle school	406 (41.81)	279 (40.20)
Senior middle school and above	99 (10.20)	65 (9.37)
Occupation (farmer)	826 (85.07)	600 (86.46)
Household size (reference group = 1–3 members)		
≥ 4 members	525 (54.07)	382 (55.04)
Chronic disease	588 (60.56)	352 (50.72)
Self-assessed health (reference group = worse)		
Normal	261 (26.88)	168 (24.21)
Better	493 (50.77)	413 (59.51)
Total	971	694

## Methods

### Definition of CHEs

In this study, we use 40 % of a household’s capacity to pay as the threshold for determining CHEs. More specifically, a household was defined as incurring CHEs if annual hospital expenditures are equal to or higher than 40 % of its capacity to pay (CTP). CTP is usually defined as pre-payment income minus basic necessity expenditure [2]. In a developing country context, given the lack of organised labour markets and the high variability of incomes over time, household consumption, or even expenditure, is generally considered to be a better measure of welfare and CTP, than is income [28]. In our study, we use total household expenditures to measure pre-payment income and food expenditures as a proxy for necessities [2, 3]. In our sensitivity analysis, we also tested CHEs as defined using other thresholds, 20 %, 30 %, 50 %, and 60 % of CTP [13].

Inpatient care is usually costly, and the majority of NCMS enrolees who incurred CHEs did so as a result of hospitalisation [3]. In this study, we included non-medical direct expenses related to seeking care, such as transport, accommodation, food, caregiving, and so on. We calculated these expenses during the lengths of stay and used income lost because of loss of working time as the caregiving expense. The out-of-pocket (OOP) payment after insurance was calculated by subtracting the total hospital expenditure from the amount that was reimbursed. CHEs were calculated by the OOP payment and non-medical direct expenses as a proportion of CTP.

### Incidence and intensity of “catastrophic” payments

We use three indices to measure CHEs under the NCMS: catastrophic payment headcount, mean catastrophic payment gap, and mean positive payment gap. Catastrophic payment headcount is defined here as the proportion of households with health expenditures that exceeded the threshold; it reflects the incidence of CHEs. The mean catastrophic payment gap is defined here as the average degree by which payments exceed the threshold; it measures both incidence and intensity of the CHEs. The mean positive payment gap is defined here as payments in excess of the threshold in households that exceed the threshold [1]; It measures the intensity of the CHEs. The formulas are as follows:

We define T as health expenditures and X as the ability to pay, which equals total minus food expenditures. Then, T/X represents the proportion of health expenditures. Let household catastrophic overshoot O_i_ equal T_i_/X_i_ minus the threshold if it is positive and zero otherwise. Define an indicator E = 1 if O_i_ > 0 and zero otherwise. Then, the catastrophic payment headcount is given by:

$$ {\mathrm{H}}_{\mathrm{cat}}=\frac{1}{\mathrm{N}}{\displaystyle {\sum}_{\mathrm{i}=1}^{\mathrm{N}}{\mathrm{E}}_{\mathrm{i}}} $$

where N is the sample size (the total number of households).

The mean catastrophic payment gap is defined as:

$$ {\mathrm{G}}_{\mathrm{cat}}=\frac{1}{\mathrm{N}}{\displaystyle {\sum}_{\mathrm{i}=1}^{\mathrm{N}}{\mathrm{O}}_{\mathrm{i}}} $$

And the mean positive payment gap is calculated as:

$$ \mathrm{M}\mathrm{P}{\mathrm{G}}_{\mathrm{cat}}={\mathrm{G}}_{\mathrm{cat}}/{\mathrm{H}}_{\mathrm{cat}} $$

### Inequality between poor and non-poor households

The Concentration Index (CI) is a common index for measuring inequality in health service [29–32]. The CI is defined as twice the area between the concentration curve and the line of equality (the 45-degree line). In this article, we use CI to judge whether poor households incurred more CHEs than did non-poor households. The CI formula is as follows:

$$ \mathrm{C}=\frac{2}{\upmu}\mathrm{c}\mathrm{o}\mathrm{v}\left(\mathrm{h},\mathrm{r}\right) $$

in which r is the rank of socioeconomic status, h is whether the household had incurred CHEs, μ is the mean CHE in the sample, and cov is the weighted covariance.

Socioeconomic status was determined by households’ per capita expenditures. We define C_E_ as the concentration index of catastrophic payment headcount and C_O_ as the CI of the catastrophic overshoot across income. The value of the concentration index can vary between −1 and +1. A positive value of C_E_ means a greater tendency for the rich to exceed the threshold, whereas a negative value indicates a greater tendency for the poor to do so. Similarly, a positive C_O_ indicates that excess payments are more likely to be concentrated among the rich households, whereas a negative value shows that the overshoots are more likely to be concentrated among the poor households. When there is no inequality, the concentration index will be zero.

### Decomposing CHE inequalities

We also follow the method proposed by Wagstaff et al. and Hosseinpoor et al. to decompose CI into its determinant variables to explain how they contribute to inequality [33, 34]. According to the decomposition, we calculate the CI of each variable, as well as the percentage of CHE contribution to the concentration index. In our analysis, CHEs are measured as a binary variable, either one or zero, depending on whether the proportion of hospitalisation expenditures exceeded the threshold of 40 %. We used the natural logarithm of the CHE odds rather than the observed CHE. A positive CHE contribution to socioeconomic inequality meant that the distribution of explanatory variable by economic status increased socioeconomic inequality (or vice versa) [35]. The formula can be written as:

$$ \mathrm{C}={\displaystyle {\sum}_{\mathrm{k}}\left(\frac{\upbeta_{\mathrm{k}}{\overline{\mathrm{x}}}_{\mathrm{k}}}{\upmu}\right)}{\mathrm{C}}_{\mathrm{k}}+\frac{\mathrm{G}{\mathrm{C}}_{\upvarepsilon}}{\upmu} $$

Where β_k_, ${\overline{\mathrm{x}}}_{\mathrm{k}}$, and C_k_ are the coefficient, mean, and concentration index of X_k_, respectively, $\frac{\upbeta_{\mathrm{k}}{\overline{\mathrm{x}}}_{\mathrm{k}}}{\upmu}$ is the elasticity of h with respect to X_k_ which is the measurement of how responsive a variable (h) is to a change in another (X_k_), and $\left(\frac{\upbeta_{\mathrm{k}}{\overline{\mathrm{x}}}_{\mathrm{k}}}{\upmu}\right){\mathrm{C}}_{\mathrm{k}}$ is the contribution to CI.

### Decomposing changes in CHE inequalities

We use Oaxaca-type decomposition to determine how much changes in inequality were attributable to changes in inequalities in the determinants [33, 34, 36]. We denote by η_kt_ the elasticity of h with respect to X_k_ at time t, the formula can be written as:

$$ {\displaystyle {\sum}_{\mathrm{k}}{\eta}_{\mathrm{k}\mathrm{t}}\left({\mathrm{C}}_{\mathrm{k}\mathrm{t}}{\textstyle \hbox{-} }{\mathrm{C}}_{\mathrm{k}\mathrm{t}{\textstyle \hbox{-} }1}\right)+{\displaystyle {\sum}_{\mathrm{k}}{\mathrm{C}}_{\mathrm{k}\mathrm{t}{\textstyle \hbox{-} }1}\left({\eta}_{\mathrm{k}\mathrm{t}}{\textstyle \hbox{-} }{\eta}_{\mathrm{k}\mathrm{t}{\textstyle \hbox{-} }1}\right)+\Delta \left(\frac{\mathrm{G}{\mathrm{C}}_{\epsilon \mathrm{t}}}{\mu_{\mathrm{t}}}\right)}} $$

This decomposition allowed us to assess the extent to which changes in CHE inequalities were due to changes in inequality in the determinants rather than changes in elasticity.

## Results

### Determinants of hospitalisation of households

It was important to study how the probability of being hospitalised developed between those two years when we adjusted for other variables that influence the probability of being hospitalised. It was found that the proportions of households that experienced a hospitalisation in 2009 and 2012 are 13.49 % and 15.56 %, respectively. Thus, a more detailed analysis was conducted of the household-level random effects (to account for unobserved heterogeneity at the household level) using a logit model to analyse the determinants of household hospitalisation. Some variables exist that are not changed over time including sex, occupation, and others. Thus, the random effect terms were analysed as those variables may influence the hospitalisation [37, 38].

The results of random-effect logit model were shown in Table 3. The year dummy variable shows an increase in the rate of hospitalisations in 2012 compared with 2009. The results also show that hospitalisation was more likely to occur in the higher socioeconomic quintiles. Having a family member aged 5 or younger and having a male as the household head increased the probability of hospitalisation. Household size equal to or greater than 4 members and members with a chronic disease also increased the probability of hospitalisation. The better the self-assessed health of the household member who reported the poorest health, the lower the probability of hospitalisation.

**Table 3:** Determinants of household hospitalisations

	Coefficient	Std.Err	*p*
Economic status (reference group = Quintile1)			
Quintile2	0.288	0.298	0.333
Quintile3	0.725	0.292	0.013
Quintile4	1.326	0.295	0.000
Quintile5	1.879	0.303	0.000
Household member ≥ 65	0.175	0.193	0.365
Household member ≤ 5	0.700	0.223	0.002
Household head			
Sex (male)	0.782	0.350	0.026
Education (reference group = below primary school level)			
Junior middle school	−0.262	0.189	0.166
Senior middle school and above	−0.337	0.325	0.299
Occupation (farmer)	0.290	0.268	0.279
Household size (reference group = 1–3 members)			
≥ 4 members	0.444	0.189	0.019
Chronic disease	0.577	0.215	0.007
Self-assessed health (reference group = worse)			
Normal	−0.484	0.213	0.023
Better	−1.091	0.236	0.000
year (2012)	0.118	0.054	0.029

### CHE and its inequality according to economic status

The incidence and intensity of CHEs are shown on Table 4, Table 5 and Table 6. Comparing the two years, after reimbursement, the incidence and the intensity was lower in 2012. The impact of the NCMS on reducing CHEs was shown to be much higher in 2012 compared to 2009.

**Table 4:** Headcount of catastrophic health expenditures (CHEs) before and after reimbursement in 2009 and 2012

Threshold	Before reimbursement (a) (%)		After reimbursement (b) (%)		Reduction by the NCMS	
					(a–b)/a (%)	
	2009	2012	2009	2012	2009	2012
20 %	61.83	74.07	54.96	52.78	11.11	28.74
30 %	45.80	62.04	42.75	39.81	6.66	35.83
40 %	31.30	46.30	26.72	26.85	14.63	42.01
50 %	22.14	28.70	19.85	14.81	10.34	48.40
60 %	16.79	22.22	12.21	8.33	27.28	62.51

**Table 5:** Mean catastrophic payment gap in catastrophic health expenditures (CHEs) before and after reimbursement in 2009 and 2012

Threshold	Before reimbursement (A) (%)		After reimbursement (B) (%)		Reduction by NCMS	
					A–B	
	2009	2012	2009	2012	2009	2012
20 %	17.62	22.42	13.79	12.49	3.83	9.93
30 %	12.43	15.78	9.06	7.90	3.37	7.88
40 %	8.33	10.52	5.82	4.47	2.51	6.05
50 %	5.69	6.89	3.52	2.39	2.17	4.50
60 %	3.71	4.35	1.91	1.18	1.80	3.17

**Table 6:** Mean positive payment gap in catastrophic health expenditures (CHEs) before and after reimbursement in 2009 and 2012

Threshold	Before reimbursement (A) (%)		After reimbursement (B) (%)		Reduction by NCMS A–B	
	2009	2012	2009	2012	2009	2012
20 %	28.50	30.27	25.09	23.66	3.41	6.60
30 %	27.14	25.44	21.19	19.84	5.95	5.59
40 %	26.61	22.72	21.78	16.65	4.83	6.07
50 %	25.70	24.01	17.73	16.14	7.97	7.87
60 %	22.10	19.58	15.64	14.17	6.45	5.41

Table 7 shows higher inequality after reimbursement compared with before. After reimbursement, the rich had a greater tendency to incur CHEs and there existed less inequality in the incidence of CHEs after reimbursement in 2012 compared with 2009. Regarding the intensity of CHEs, the overshoot tended to be greater among the more wealthy households in 2009, but it tended to be greater among the poor in 2012.

**Table 7:** Concentration Index (CI) for weighted headcount and gap at the 40 % threshold in 2009 and 2012

Year	Before reimbursement		After reimbursement	
	2009	2012	2009	2012
C_E_	0.031	−0.011	0.073	0.021
C_O_	0.075	−0.028	0.130	−0.203

### Decomposition of socioeconomic inequality in catastrophic health care expenditures

Table 8 shows how the socioeconomic inequality in CHEs can be explained through decomposition of the concentration index in the 2009 and 2012 at the threshold of 40 %. The CIs of the variables having household members older than 65 years or younger than 5 years of age, larger household size and higher reimbursement are negative in both years, which means these variables were concentrated among people with lower economic status. In contrast, stays at institutions above the county level or for longer than 15 days had a positive concentration index in both years, indicating higher use of these services among the rich.

**Table 8:** Decomposition analysis of concentration index of catastrophic health care expenditure in 2009 and 2012

	Coefficient		Mean		Elasticity		Concentration index (CI)		Contribution to CI	
	2009	2012	2009	2012	2009	2012	2009	2012	2009	2012
Economic status (reference group = Quintile1)										
Quintile2	−0.089	−0.068	0.198	0.194	−0.066	−0.049	−0.392	−0.402	0.026	0.020
Quintile3	−0.094	−0.162	0.198	0.204	−0.070	−0.123	0.008	0.000	−0.001	0.000
Quintile4	−0.103	−0.165	0.198	0.194	−0.077	−0.12	0.408	0.402	−0.031	−0.048
Quintile5	−0.071	−0.119	0.198	0.204	−0.053	−0.090	0.808	0.804	−0.043	−0.073
Household member ≥ 65	0.274*	−0.009	0.359	0.370	0.368	−0.012	−0.067	−0.037	−0.025	0.000
Household member ≤ 5	0.172	0.010	0.244	0.222	0.157	0.008	−0.259	−0.192	−0.041	−0.002
Household head										
Sex (male)	−0.040	−0.107	0.947	0.935	−0.141	−0.373	−0.026	−0.025	0.004	0.009
Education level (reference group = below primary school level)										
Junior middle school	0.152	−0.031	0.382	0.352	0.217	−0.040	−0.042	0.025	−0.009	−0.001
Senior middle school and above	−0.049	−0.059	0.076	0.074	−0.014	−0.016	0.398	−0.112	−0.006	0.002
Occupation (farmer)	−0.148	0.033	0.863	0.926	−0.478	0.115	0.006	0.001	−0.003	0.000
Household size (reference group = 1–3 members)										
≥ 4 members	−0.348**	−0.145	0.580	0.676	−0.756	−0.366	−0.115	−0.092	0.087	0.034
Real reimbursement	−0.067	−0.857**	0.179	0.331	−0.045	−1.055	−0.025	−0.028	0.001	0.030
Length of stay (reference group = fewer than 8 days)										
8–14 days	−0.003	−0.050	0.267	0.287	−0.003	−0.053	0.070	−0.238	0.000	0.013
≥ 15 days	0.471**	0.221*	0.328	0.398	0.579	0.328	0.146	0.257	0.085	0.084
Inpatient institution (reference group = town level)										
County level	0.080	0.111	0.519	0.537	0.155	0.222	0.038	0.038	0.006	0.008
County above	0.487**	0.365	0.183	0.213	0.334	0.290	0.038	0.183	0.013	0.053
Self-assessed health (reference group = worse)										
Normal	−0.047	−0.035	0.305	0.324	−0.053	−0.042	−0.178	−0.040	0.009	0.002
Better	−0.089	−0.062	0.305	0.380	−0.102	−0.088	0.156	−0.089	−0.016	0.008

*Statistically significant (*P* <0.05), **Highly statistically significant (*P* <0.01)

The column for contribution to CI shows the absolute contribution of each determinant variable to socioeconomic inequality. The results show that the contributions of household size and hospitalisation-related variables, such as real reimbursement, length of stay, and inpatient institutions were positive, which means these variables increased inequality in terms of higher CHEs among the rich. Economic status had a negative value. Other variables had small contributions to the inequality in both years.

### Decomposing changes in CHE inequalities between 2009 and 2012

Our results indicate that from 2009 to 2012, inequality in CHEs after reimbursement decreased by 0.052. We then decompose this reduction in CHE inequality to seek contributing factors. It can be observed from Table 9 that the changes in inequality were not attribute to the changes in the variables, especially real reimbursement percentage, and choosing an institution above the county level because their contributions were negative. It is important to note that the changes of reimbursement percentage were mainly due to changes in elasticity rather than inequality. However, the contribution to the inequality in the CHE variables, in particular economic status and household size, tended to be positive, indicating that the poor were more likely to incur CHEs than were the rich after reimbursement from 2009 to 2012.

**Table 9:** Oaxaca-type decomposition for changes in inequality, 2009–2012

	2009–2012			
	∆C*η	∆η*C	Total	%
Economic status (reference group = Quintile1)				
Quintile2	0.000	−0.007	−0.006	11.873
Quintile3	0.001	0.000	0.001	−1.077
Quintile4	0.001	−0.018	−0.017	32.354
Quintile5	0.000	−0.030	−0.030	56.800
Household member ≥ 65	0.000	0.025	0.025	−48.308
Household member ≤ 5	0.001	0.039	0.039	−75.207
Household head				
Sex (male)	0.000	0.006	0.006	−10.883
Education level (reference group = below primary school level)				
Junior middle school	−0.003	0.011	0.008	−15.580
Senior middle school and above	0.008	−0.001	0.007	−14.162
Occupation (farmer)	−0.001	0.004	0.003	−5.737
Household size (reference group = 1–3 members)				
≥ 4 members	−0.008	−0.045	−0.053	102.438
Real reimbursement	0.003	0.025	0.028	−54.644
Length of stay (reference group = fewer than 8 days)				
8–14 days	0.016	−0.004	0.013	−24.662
≥ 15 days	0.036	−0.037	0.000	0.578
Inpatient institution (reference group = town level)				
County level	0.000	0.003	0.003	−4.911
County above	0.042	−0.002	0.040	−77.572
Self-assessed health (reference group = worse)				
Normal	−0.006	−0.002	−0.008	14.912
Better	0.022	0.002	0.024	−45.662

## Discussion

Using an individual-level survey that was conducted in Shandong province in 2009 and 2012, this study compared the CHEs incurred from hospitalisation before and after NCMS reimbursement in rural areas of China. The focus was to analyse the impact of the NCMS on reducing the financial burden for rural residents. The research also used a concentration index and decomposition to analyse socioeconomic inequality in CHEs.

We checked the change in rate of hospitalisation in 2012 compared with 2009. After we adjusted for potential contributing factors, a positive time trend was found in the rate of hospitalisation. Hospitalisation was more likely to occur in the higher economic status quintiles and among individuals with a chronic disease. The reason could be that the payment capacity was higher within this the higher income group, and that these individuals and those who suffered from a chronic disease also had greater health service demands; the individuals who were ≤5 years old were the high-risk group, with higher disease incidence [26], and larger household size may also have been related to a higher probability of suffering disease. This study also found that the households with a male head increased the probability of hospitalisation. This can be explained by the fact that the male head of a household is usually more likely to be able to provide better economic circumstances for a family, by being the main labour force of the family and the provider of the main source of income [39]. Better self-assessed health status lowered the probability of hospitalisation, because of the lower demand of health service. These results were consistent with those from other studies [25, 40].

Importantly, we found that NCMS reimbursement reduced both the incidence and intensity of CHEs, and this impact was stronger in 2012 compared with 2009. This is likely related to the health care reform that was implemented in 2009, which contained a number of policies to strengthen government financial support to the NCMS and to expand its coverage. However, in spite of these improvements, the occurrence of CHEs remained high in our sample, indicating that the impact of the NCMS on alleviating the financial burden of rural residents was still limited. A previous study also found that the NCMS reimbursement comprised the bulk of the health expenditures and that the impact on the household of CHEs was limited [16].

In both 2009 and 2012, the rich had a greater tendency to incur CHEs than did the poor after reimbursement, and more rich people incurred CHEs after reimbursement compared with before. Another previous study that estimated the impact of the NCMS on CHEs also demonstrated this point [41]. A possible explanation of this result is that the rich were more likely to stay in hospital for longer than two weeks. In addition, the rich had a greater tendency to choose county-level institutions; and according to the NCMS policy, the higher the institution level, the lower the reimbursement rates. Another possible explanation is that the poor are more cautious than their rich counterparts in health spending in order to avoid CHEs, or the poor may simply not be able to pay for the upfront medical bills that are likely to induce CHEs due to the reimbursement procedure that the patients should pay all their medical bills before leaving hospital. This inequality was reduced in 2012.

Our results indicate that household size and hospitalisation-related variables such as real reimbursement, length of stay, and inpatient institutions tended to increase inequality in CHEs. Economic status caused higher CHEs among the poor. One other study also found that household income was inversely associated with poverty caused by health expenditures [24]. The changes in inequality between 2009 and 2012 show a trend that fewer rich people having CHEs in 2012 compared with 2009. It appears that the new health care reform had no effect on reducing the economic burden among the poor, but this study found that this attributed to the changes in economic status and household size because their contributions to the changes in inequality were positive. The negative contribution of reimbursement to the change of inequality indicated that the poor were less likely to incur CHEs than the rich after reimbursement from 2009 to 2012. This was the finding although NCMS policies under the new health care reform increased the reimbursement rate among the poor, but the effect was still limited.

The policy implications from our results are four-fold. First, healthcare reform policies in China that aim to reduce CHEs must continue to emphasise improving reimbursement, increasing subsidies, and containing healthcare costs, especially inpatient costs. Second, the key to reducing the incidence and inequality in catastrophic healthcare expenditures in China lies in reducing poverty, decreasing the gap between poor and rich, and continuing to improve the Medical Aid system, which can play a key role in giving extra financial assistance to those who are the poorest. This is consistent with findings from other studies [42, 43]. Third, cost containment policies in China should pay attention to length of hospital stay and aim to reduce unnecessary hospitalisations while maintaining quality of care. Fourth, proper incentives and gatekeeping mechanisms should be implemented to ensure that patients seek care at the appropriate hospital levels. Overuse of county-level institutions may lead to waste of resources.

Our study has a number of limitations. First, due to the limitations in the survey data, we only analysed the inpatient health expenditures for households that experienced a hospitalisation in the previous year. This could possibly underestimate the incidence of CHEs. Second, our sample was limited to three representative counties in Jinan city in the Shandong province, and thus was not nationally representative. Thus, whether the conclusions drawn from this study are applicable to the whole country is uncertain. Lastly, we used a uniform threshold in defining CHEs, regardless of the household income level. However, rich households may have a greater capacity to pay and may be able to spend more on health care without affecting their consumption of necessities. It is likely more appropriate to use a higher threshold for rich households when defining CHEs. Future research should attempt to overcome these limitations and seek nationally representative data to evaluate the evolution of the impacts of the NCMS.

## Conclusions

Our study found that the impact of the NCMS on alleviating the financial burden of rural residents was still limited and there were inequalities in the CHEs. The rich were more likely to incur CHEs than were the poor after reimbursement. This inequality decreased in 2012, indicating that the change in NCMS policies might have reduced the difference in care-seeking behaviour between the rich and poor. With the decomposition of the CI, our study indicated that household size and hospitalisation-related variables tended to increase inequalities in CHEs. The Oaxaca decomposition analysis, based on the decomposition of the CI, suggested that the changes in inequality between 2009 and 2012 show a trend that the fewer rich people having CHEs in 2012 compared with 2009, and this attributed to the changes in economic status and household size rather than reimbursement levels. Policies should still emphasise improving reimbursement and socioeconomic status and reducing unnecessary hospitalisation, by reducing poverty and the gap between poor and rich; continuing to improve the Medical Aid system; maintaining quality of care; and establishing proper incentive and gatekeeping mechanisms.

## Abbreviations

CHEs: Catastrophic health expenditures
CI: Concentration index
CMS: Cooperative medical scheme
CTP: Capacity to pay
NCMS: New cooperative medical scheme
OOP: Out-of-pocket
